# 2-(1*H*-Benzotriazol-1-yl)-1-(4-ethyl­benzo­yl)ethyl 2-chloro­benzoate

**DOI:** 10.1107/S1600536809031912

**Published:** 2009-08-15

**Authors:** Wu-Lan Zeng, Fang-Fang Jian

**Affiliations:** a Microscale Science Institute, Department of Chemistry and Chemical Engineering, Weifang University, Weifang 261061, People’s Republic of China; b Microscale Science Institute, Weifang University, Weifang 261061, People’s Republic of China

## Abstract

In the crystal structure of the title compound, C_24_H_20_ClN_3_O_3_, weak inter­molecular C—H⋯O hydrogen bonds link the mol­ecules into chains extended along the *a* axis. The crystal studied was found to be an inversion twin.

## Related literature

For background to the pharmacological activity of 1*H*-benzotriazole and its derivative, see Chen & Wu (2005 ▶). For reference structural data, see: Allen *et al.* (1987 ▶).
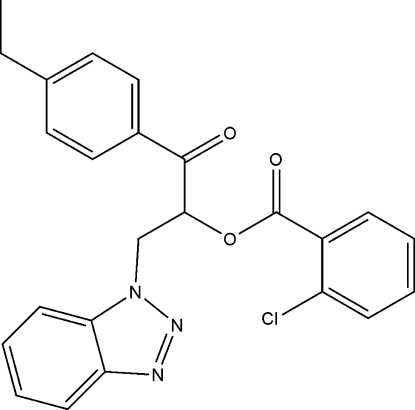


## Experimental

### 

#### Crystal data

C_24_H_20_ClN_3_O_3_
*M* *_r_* = 433.88
Orthorhombic, 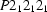

*a* = 9.433 (2) Å
*b* = 14.824 (4) Å
*c* = 15.239 (4) Å
*V* = 2131.0 (10) Å^3^
*Z* = 4
Mo *K*α radiation
μ = 0.21 mm^−1^
*T* = 293 K
0.15 × 0.12 × 0.10 mm

#### Data collection

Bruker SMART CCD diffractometer
Absorption correction: none
13991 measured reflections
5212 independent reflections
2174 reflections with *I* > 2σ(*I*)
*R*_int_ = 0.078

#### Refinement

*R*[*F* ^2^ > 2σ(*F* ^2^)] = 0.057
*wR*(*F* ^2^) = 0.158
*S* = 0.91
5212 reflections
281 parameters
H-atom parameters constrained
Δρ_max_ = 0.22 e Å^−3^
Δρ_min_ = −0.20 e Å^−3^
Absolute structure: Flack (1983 ▶), 2244 Friedel pairs
Flack parameter: 0.57 (12)

### 

Data collection: *SMART* (Bruker, 1997 ▶); cell refinement: *SAINT* (Bruker, 1997 ▶); data reduction: *SAINT*; program(s) used to solve structure: *SHELXS97* (Sheldrick, 2008 ▶); program(s) used to refine structure: *SHELXL97* (Sheldrick, 2008 ▶); molecular graphics: *SHELXTL* (Sheldrick, 2008 ▶); software used to prepare material for publication: *SHELXTL*.

## Supplementary Material

Crystal structure: contains datablocks global, I. DOI: 10.1107/S1600536809031912/hb5022sup1.cif

Structure factors: contains datablocks I. DOI: 10.1107/S1600536809031912/hb5022Isup2.hkl

Additional supplementary materials:  crystallographic information; 3D view; checkCIF report

## Figures and Tables

**Table 1:** Hydrogen-bond geometry (Å, °)

*D*—H⋯*A*	*D*—H	H⋯*A*	*D*⋯*A*	*D*—H⋯*A*
C7—H7*B*⋯O3^i^	0.97	2.51	3.162 (4)	125
C8—H8*A*⋯O2^ii^	0.98	2.42	3.397 (5)	171
C11—H11*A*⋯O2^ii^	0.93	2.56	3.431 (5)	155

Symmetry codes: (i) 
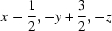
; (ii) 
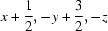
.

**Fig. 1.**
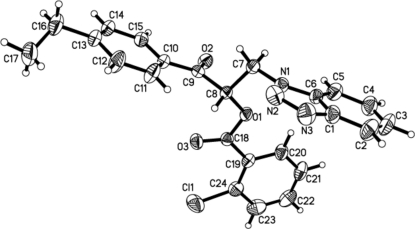
The molecular structure of (I), drawn with 30% probability ellipsoids (arbitrary spheres for the H atoms).

## supplementary crystallographic information

### Comment

1*H*-Benzotriazole and its derivatives exhibit a broad spectrum of pharmacological activities such as antifungal, antitumor and antineoplastic activities (Chen & Wu, 2005). We report here the synthesis and structure of the title compound, (I) (Fig. 1), as part of our ongoing studies on new benzotriazole compounds with higher bioactivity.

All the bond lengths and angles in (I) are within their normal ranges (Allen *et al.*, 1987). The benzotriazole ring system is essentially planar, with a dihedral angle of 1.97 (1)° between the triazole ring (atoms N1—N3/C1/C6) and the C1—C6 benzene ring. The dihedral angles between the mean planes of the benzotriazole system and the C10—C15 and C19—C24 aromatic rings are 9.25 (1)° and 87.55 (1)°, respectively. The dihedral angle between rings C10—C15 and C19—C24 is 85.25 (2)°. Molecule (I) is chiral: atom C8 has *S* configuration, but refinement showed the crystal to be a racemic twin.

### Experimental

Bromine (3.2 g, 0.02 mol) was added dropwise to a solution of 3-(1*H*-benzo[*d*][1,2,3]triazol-1-yl)-1-(4-ethylphenyl)propan-1-one (5.58 g, 0.02 mol) and sodium acetate (1.6 g, 0.02 mol) in acetic acid (50 ml). The reaction proceeded for 7 h. Water (50 ml) and chloroform (20 ml) were then added. The organic layer was washed successively with saturated sodium bicarbonate solution and brine, dried over anhydrous magnesium sulfate and the chloroform solution filtered. It was cooled with ice-water, and then an acetone solution (10 ml) of 2-chlorobenzoic acid (3.1 g, 0.02 mol) and triethylamine (2.8 ml) was added. The mixture was stirred with ice-water for 6 h. The solution was then filtered and concentrated.  Colourless blocks of (I) were obtained by slow evaporation of an petroleum aether-ethylacetate (3:1 *v*/*v*) solution at room temperature over a period of one week.

### Refinement

The H atoms were geometrically placed (C—H = 0.93–0.97 Å), and refined as riding with *U*_iso_(H) = 1.2*U*_eq_(C) or 1.5*U*_eq_(methyl C).

### Crystal data

**Table:**

C_24_H_20_ClN_3_O_3_	*F*(000) = 904
*M**_r_* = 433.88	*D*_x_ = 1.352 Mg m^−^^3^
Orthorhombic, *P*2_1_2_1_2_1_	Mo *K*α radiation, λ = 0.71073 Å
Hall symbol: P 2ac 2ab	Cell parameters from 5212 reflections
*a* = 9.433 (2) Å	θ = 1.9–28.3°
*b* = 14.824 (4) Å	µ = 0.21 mm^−^^1^
*c* = 15.239 (4) Å	*T* = 293 K
*V* = 2131.0 (10) Å^3^	Block, colourless
*Z* = 4	0.15 × 0.12 × 0.10 mm

### Data collection

**Table:**

Bruker SMART CCD diffractometer	2174 reflections with *I* > 2σ(*I*)
Radiation source: fine-focus sealed tube	*R*_int_ = 0.078
graphite	θ_max_ = 28.3°, θ_min_ = 1.9°
ω scans	*h* = −12→12
13991 measured reflections	*k* = −19→17
5212 independent reflections	*l* = −20→13

### Refinement

**Table:**

Refinement on *F*^2^	Hydrogen site location: inferred from neighbouring sites
Least-squares matrix: full	H-atom parameters constrained
*R*[*F*^2^ > 2σ(*F*^2^)] = 0.057	*w* = 1/[σ^2^(*F*_o_^2^) + (0.0626*P*)^2^] where *P* = (*F*_o_^2^ + 2*F*_c_^2^)/3
*wR*(*F*^2^) = 0.158	(Δ/σ)_max_ < 0.001
*S* = 0.91	Δρ_max_ = 0.22 e Å^−^^3^
5212 reflections	Δρ_min_ = −0.20 e Å^−^^3^
281 parameters	Extinction correction: *SHELXL97* (Sheldrick, 2008), Fc^*^=kFc[1+0.001xFc^2^λ^3^/sin(2θ)]^-1/4^
0 restraints	Extinction coefficient: 0.0034 (10)
Primary atom site location: structure-invariant direct methods	Absolute structure: Flack (1983), 2214 Friedel pairs
Secondary atom site location: difference Fourier map	Flack parameter: 0.57 (12)

### Special details

**Table:**

Geometry. All e.s.d.'s (except the e.s.d. in the dihedral angle between two l.s. planes) are estimated using the full covariance matrix. The cell e.s.d.'s are taken into account individually in the estimation of e.s.d.'s in distances, angles and torsion angles; correlations between e.s.d.'s in cell parameters are only used when they are defined by crystal symmetry. An approximate (isotropic) treatment of cell e.s.d.'s is used for estimating e.s.d.'s involving l.s. planes.
Refinement. Refinement of *F*^2^ against ALL reflections. The weighted *R*-factor *wR* and goodness of fit *S* are based on *F*^2^, conventional *R*-factors *R* are based on *F*, with *F* set to zero for negative *F*^2^. The threshold expression of *F*^2^ > σ(*F*^2^) is used only for calculating *R*-factors(gt) *etc*. and is not relevant to the choice of reflections for refinement. *R*-factors based on *F*^2^ are statistically about twice as large as those based on *F*, and *R*- factors based on ALL data will be even larger.

### Fractional atomic coordinates and isotropic or equivalent isotropic displacement parameters (Å^2^)

**Table:**

	*x*	*y*	*z*	*U*_iso_*/*U*_eq_	
Cl1	0.15025 (14)	0.62148 (8)	0.24883 (9)	0.0933 (5)	
O1	−0.1687 (2)	0.61438 (14)	0.03013 (16)	0.0470 (6)	
O2	−0.3655 (3)	0.73734 (15)	0.08575 (17)	0.0573 (7)	
O3	−0.0410 (3)	0.69848 (17)	0.12131 (18)	0.0572 (7)	
N1	−0.2042 (3)	0.6104 (2)	−0.15765 (19)	0.0497 (8)	
N2	−0.1122 (4)	0.6446 (2)	−0.2173 (2)	0.0660 (10)	
N3	−0.0555 (4)	0.5770 (3)	−0.2603 (2)	0.0788 (11)	
C1	−0.1124 (5)	0.4984 (3)	−0.2287 (3)	0.0627 (12)	
C2	−0.0891 (5)	0.4096 (3)	−0.2526 (3)	0.0803 (14)	
H2B	−0.0234	0.3952	−0.2958	0.096*	
C3	−0.1639 (7)	0.3446 (3)	−0.2116 (3)	0.0887 (17)	
H3B	−0.1497	0.2846	−0.2270	0.106*	
C4	−0.2615 (6)	0.3655 (3)	−0.1468 (3)	0.0806 (14)	
H4A	−0.3124	0.3190	−0.1206	0.097*	
C5	−0.2858 (5)	0.4534 (3)	−0.1199 (3)	0.0669 (12)	
H5A	−0.3507	0.4675	−0.0760	0.080*	
C6	−0.2070 (4)	0.5187 (3)	−0.1626 (3)	0.0502 (10)	
C7	−0.2789 (4)	0.6705 (2)	−0.0990 (2)	0.0508 (10)	
H7A	−0.3658	0.6415	−0.0799	0.061*	
H7B	−0.3047	0.7248	−0.1308	0.061*	
C8	−0.1931 (4)	0.6963 (2)	−0.0194 (2)	0.0430 (9)	
H8A	−0.1022	0.7221	−0.0379	0.052*	
C9	−0.2747 (4)	0.7649 (2)	0.0346 (3)	0.0433 (9)	
C10	−0.2528 (4)	0.8626 (2)	0.0197 (2)	0.0433 (9)	
C11	−0.1516 (5)	0.8960 (3)	−0.0356 (3)	0.0705 (13)	
H11A	−0.0911	0.8566	−0.0649	0.085*	
C12	−0.1383 (5)	0.9887 (3)	−0.0485 (3)	0.0804 (15)	
H12A	−0.0683	1.0103	−0.0860	0.097*	
C13	−0.2257 (5)	1.0486 (2)	−0.0072 (3)	0.0656 (12)	
C14	−0.3253 (5)	1.0147 (3)	0.0490 (3)	0.0650 (13)	
H14A	−0.3849	1.0542	0.0789	0.078*	
C15	−0.3386 (4)	0.9232 (2)	0.0622 (3)	0.0545 (10)	
H15A	−0.4073	0.9020	0.1009	0.065*	
C16	−0.2116 (6)	1.1492 (3)	−0.0229 (4)	0.0990 (18)	
H16A	−0.2517	1.1628	−0.0800	0.119*	
H16B	−0.2687	1.1803	0.0205	0.119*	
C17	−0.0676 (6)	1.1865 (3)	−0.0198 (4)	0.109 (2)	
H17A	−0.0709	1.2501	−0.0311	0.164*	
H17B	−0.0099	1.1575	−0.0634	0.164*	
H17C	−0.0276	1.1761	0.0373	0.164*	
C18	−0.0826 (4)	0.6257 (2)	0.1005 (2)	0.0443 (9)	
C19	−0.0495 (4)	0.5386 (2)	0.1438 (2)	0.0459 (10)	
C20	−0.1196 (5)	0.4611 (2)	0.1187 (3)	0.0602 (11)	
H20A	−0.1879	0.4645	0.0748	0.072*	
C21	−0.0906 (6)	0.3783 (3)	0.1573 (3)	0.0742 (14)	
H21A	−0.1399	0.3270	0.1397	0.089*	
C22	0.0106 (6)	0.3724 (3)	0.2212 (3)	0.0772 (14)	
H22A	0.0306	0.3170	0.2471	0.093*	
C23	0.0820 (5)	0.4473 (3)	0.2466 (3)	0.0840 (15)	
H23A	0.1517	0.4428	0.2895	0.101*	
C24	0.0519 (5)	0.5315 (3)	0.2090 (3)	0.0589 (11)	

### Atomic displacement parameters (Å^2^)

**Table:**

	*U*^11^	*U*^22^	*U*^33^	*U*^12^	*U*^13^	*U*^23^
Cl1	0.1010 (9)	0.0888 (9)	0.0902 (9)	−0.0149 (8)	−0.0451 (8)	0.0174 (8)
O1	0.0570 (15)	0.0334 (13)	0.0505 (15)	0.0011 (12)	−0.0078 (13)	0.0052 (12)
O2	0.0568 (17)	0.0499 (15)	0.0652 (19)	0.0002 (14)	0.0106 (16)	0.0067 (14)
O3	0.0709 (18)	0.0426 (15)	0.0583 (17)	−0.0093 (14)	−0.0145 (15)	0.0040 (14)
N1	0.057 (2)	0.0464 (19)	0.0452 (18)	0.0041 (16)	−0.0044 (17)	−0.0047 (16)
N2	0.074 (2)	0.073 (2)	0.051 (2)	−0.011 (2)	0.004 (2)	0.001 (2)
N3	0.091 (3)	0.086 (3)	0.060 (2)	−0.002 (2)	0.016 (2)	−0.015 (2)
C1	0.078 (3)	0.059 (3)	0.052 (3)	0.005 (3)	−0.005 (2)	−0.011 (2)
C2	0.103 (4)	0.078 (3)	0.060 (3)	0.020 (3)	0.019 (3)	−0.011 (3)
C3	0.131 (5)	0.060 (3)	0.075 (3)	0.034 (3)	0.003 (4)	−0.015 (3)
C4	0.108 (4)	0.051 (3)	0.083 (3)	0.006 (3)	−0.014 (3)	−0.001 (3)
C5	0.084 (3)	0.058 (3)	0.059 (3)	0.010 (2)	0.000 (3)	0.001 (2)
C6	0.058 (3)	0.046 (2)	0.046 (2)	0.009 (2)	−0.009 (2)	−0.008 (2)
C7	0.057 (2)	0.045 (2)	0.051 (2)	0.0063 (19)	−0.008 (2)	−0.0040 (19)
C8	0.049 (2)	0.0317 (18)	0.049 (2)	0.0025 (16)	−0.0073 (19)	0.0034 (18)
C9	0.039 (2)	0.046 (2)	0.044 (2)	−0.0013 (18)	−0.006 (2)	−0.0013 (19)
C10	0.049 (2)	0.036 (2)	0.045 (2)	0.0026 (18)	0.0024 (19)	0.0004 (18)
C11	0.088 (3)	0.040 (2)	0.084 (3)	0.009 (2)	0.022 (3)	0.007 (2)
C12	0.094 (4)	0.044 (3)	0.104 (4)	0.000 (3)	0.037 (3)	0.013 (3)
C13	0.069 (3)	0.037 (2)	0.091 (3)	0.003 (2)	−0.009 (3)	−0.002 (2)
C14	0.063 (3)	0.044 (3)	0.088 (3)	0.011 (2)	−0.004 (3)	−0.014 (2)
C15	0.053 (2)	0.050 (2)	0.061 (2)	0.000 (2)	0.001 (2)	−0.004 (2)
C16	0.107 (4)	0.039 (2)	0.151 (5)	−0.004 (3)	−0.003 (4)	0.013 (3)
C17	0.131 (5)	0.053 (3)	0.144 (5)	−0.018 (3)	−0.027 (4)	0.011 (3)
C18	0.044 (2)	0.042 (2)	0.046 (2)	−0.0026 (19)	0.0019 (19)	0.000 (2)
C19	0.057 (2)	0.042 (2)	0.039 (2)	0.007 (2)	0.0058 (19)	0.0021 (18)
C20	0.084 (3)	0.044 (2)	0.053 (2)	0.002 (2)	−0.009 (2)	0.001 (2)
C21	0.109 (4)	0.037 (2)	0.076 (3)	0.000 (3)	0.013 (3)	0.003 (2)
C22	0.101 (4)	0.056 (3)	0.074 (3)	0.021 (3)	0.017 (3)	0.027 (3)
C23	0.092 (4)	0.085 (3)	0.075 (3)	0.019 (3)	−0.014 (3)	0.029 (3)
C24	0.068 (3)	0.054 (2)	0.054 (3)	0.005 (2)	−0.002 (2)	0.012 (2)

### Geometric parameters (Å, °)

**Table:**

Cl1—C24	1.734 (4)	C10—C15	1.373 (5)
O1—C18	1.355 (4)	C11—C12	1.394 (5)
O1—C8	1.448 (4)	C11—H11A	0.9300
O2—C9	1.228 (4)	C12—C13	1.364 (6)
O3—C18	1.191 (4)	C12—H12A	0.9300
N1—N2	1.355 (4)	C13—C14	1.368 (6)
N1—C6	1.361 (4)	C13—C16	1.516 (5)
N1—C7	1.445 (4)	C14—C15	1.378 (5)
N2—N3	1.312 (4)	C14—H14A	0.9300
N3—C1	1.371 (5)	C15—H15A	0.9300
C1—C6	1.380 (6)	C16—C17	1.468 (7)
C1—C2	1.382 (5)	C16—H16A	0.9700
C2—C3	1.348 (6)	C16—H16B	0.9700
C2—H2B	0.9300	C17—H17A	0.9600
C3—C4	1.385 (7)	C17—H17B	0.9600
C3—H3B	0.9300	C17—H17C	0.9600
C4—C5	1.385 (6)	C18—C19	1.483 (5)
C4—H4A	0.9300	C19—C20	1.379 (5)
C5—C6	1.383 (5)	C19—C24	1.383 (6)
C5—H5A	0.9300	C20—C21	1.389 (5)
C7—C8	1.508 (5)	C20—H20A	0.9300
C7—H7A	0.9700	C21—C22	1.367 (7)
C7—H7B	0.9700	C21—H21A	0.9300
C8—C9	1.518 (5)	C22—C23	1.355 (6)
C8—H8A	0.9800	C22—H22A	0.9300
C9—C10	1.480 (5)	C23—C24	1.403 (6)
C10—C11	1.367 (5)	C23—H23A	0.9300
			
C18—O1—C8	113.8 (2)	C13—C12—C11	121.5 (4)
N2—N1—C6	110.5 (3)	C13—C12—H12A	119.2
N2—N1—C7	119.8 (3)	C11—C12—H12A	119.2
C6—N1—C7	129.8 (3)	C12—C13—C14	117.7 (4)
N3—N2—N1	108.1 (3)	C12—C13—C16	121.0 (5)
N2—N3—C1	108.3 (3)	C14—C13—C16	121.3 (4)
N3—C1—C6	108.9 (4)	C13—C14—C15	121.1 (4)
N3—C1—C2	130.9 (4)	C13—C14—H14A	119.5
C6—C1—C2	120.2 (4)	C15—C14—H14A	119.5
C3—C2—C1	118.4 (4)	C10—C15—C14	121.5 (4)
C3—C2—H2B	120.8	C10—C15—H15A	119.3
C1—C2—H2B	120.8	C14—C15—H15A	119.3
C2—C3—C4	121.2 (4)	C17—C16—C13	116.5 (4)
C2—C3—H3B	119.4	C17—C16—H16A	108.2
C4—C3—H3B	119.4	C13—C16—H16A	108.2
C5—C4—C3	122.1 (5)	C17—C16—H16B	108.2
C5—C4—H4A	118.9	C13—C16—H16B	108.2
C3—C4—H4A	118.9	H16A—C16—H16B	107.3
C6—C5—C4	115.5 (4)	C16—C17—H17A	109.5
C6—C5—H5A	122.3	C16—C17—H17B	109.5
C4—C5—H5A	122.3	H17A—C17—H17B	109.5
N1—C6—C1	104.2 (4)	C16—C17—H17C	109.5
N1—C6—C5	133.1 (4)	H17A—C17—H17C	109.5
C1—C6—C5	122.6 (4)	H17B—C17—H17C	109.5
N1—C7—C8	113.0 (3)	O3—C18—O1	121.4 (3)
N1—C7—H7A	109.0	O3—C18—C19	126.9 (4)
C8—C7—H7A	109.0	O1—C18—C19	111.7 (3)
N1—C7—H7B	109.0	C20—C19—C24	117.9 (3)
C8—C7—H7B	109.0	C20—C19—C18	120.0 (4)
H7A—C7—H7B	107.8	C24—C19—C18	122.1 (3)
O1—C8—C7	107.0 (3)	C19—C20—C21	121.6 (4)
O1—C8—C9	111.0 (3)	C19—C20—H20A	119.2
C7—C8—C9	109.5 (3)	C21—C20—H20A	119.2
O1—C8—H8A	109.8	C22—C21—C20	119.7 (4)
C7—C8—H8A	109.8	C22—C21—H21A	120.1
C9—C8—H8A	109.8	C20—C21—H21A	120.1
O2—C9—C10	121.3 (3)	C23—C22—C21	119.9 (4)
O2—C9—C8	118.4 (3)	C23—C22—H22A	120.0
C10—C9—C8	120.1 (3)	C21—C22—H22A	120.0
C11—C10—C15	117.8 (3)	C22—C23—C24	120.8 (4)
C11—C10—C9	123.1 (3)	C22—C23—H23A	119.6
C15—C10—C9	119.1 (3)	C24—C23—H23A	119.6
C10—C11—C12	120.4 (4)	C19—C24—C23	120.1 (4)
C10—C11—H11A	119.8	C19—C24—Cl1	124.3 (3)
C12—C11—H11A	119.8	C23—C24—Cl1	115.7 (4)

### Hydrogen-bond geometry (Å, °)

**Table:**

*D*—H···*A*	*D*—H	H···*A*	*D*···*A*	*D*—H···*A*
C7—H7B···O3^i^	0.97	2.51	3.162 (4)	125
C8—H8A···O2^ii^	0.98	2.42	3.397 (5)	171
C11—H11A···O2^ii^	0.93	2.56	3.431 (5)	155

Symmetry codes: (i) *x*−1/2, −*y*+3/2, −*z*; (ii) *x*+1/2, −*y*+3/2, −*z*.
